# Supplementary figures and images for: Effect of Shipping on the Microbiome of Donor Mice Used to Reconstitute Germ-Free Recipients
Source: Gut Microbes Rep. 2024 Jun 28;1(1):2363858. doi: 10.1080/29933935.2024.2363858 (PMC11423901; doi:10.1080/29933935.2024.2363858)

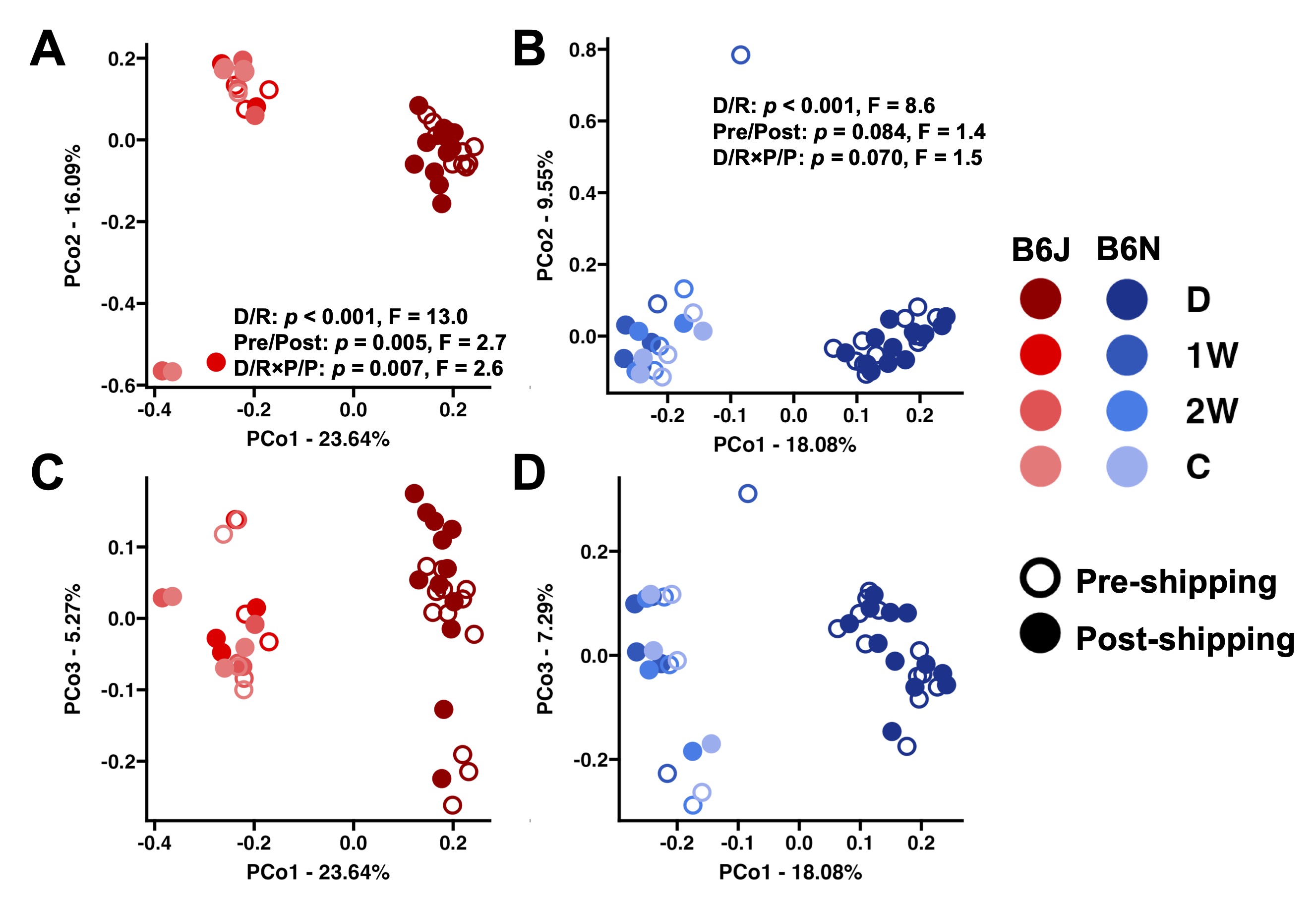

Supplement: Supplemental Material [file KGMR_A_2363858_SM6292.zip › figures/Figure3.jpg]

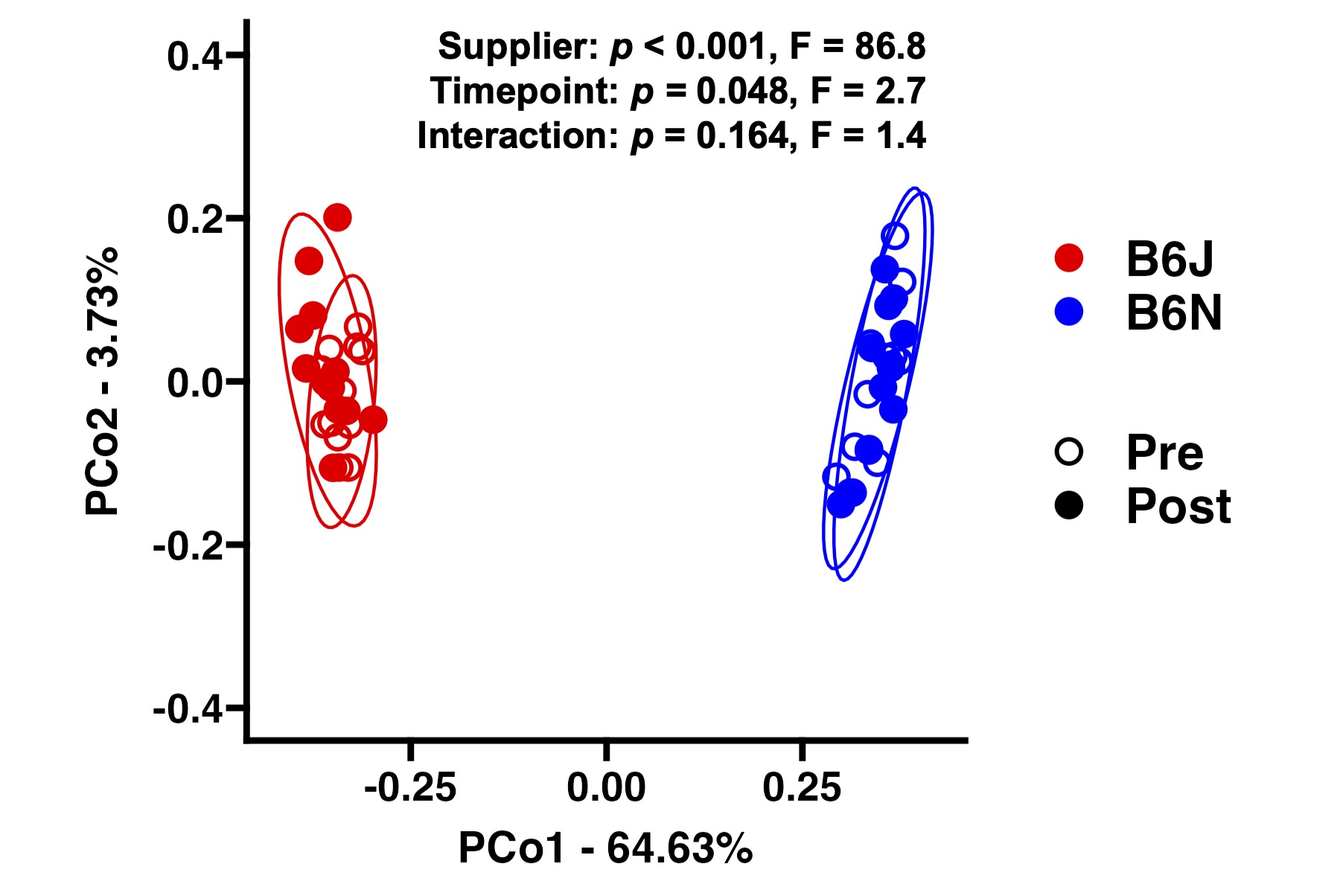

Supplement: Supplemental Material [file KGMR_A_2363858_SM6292.zip › figures/FigureS1.jpg]

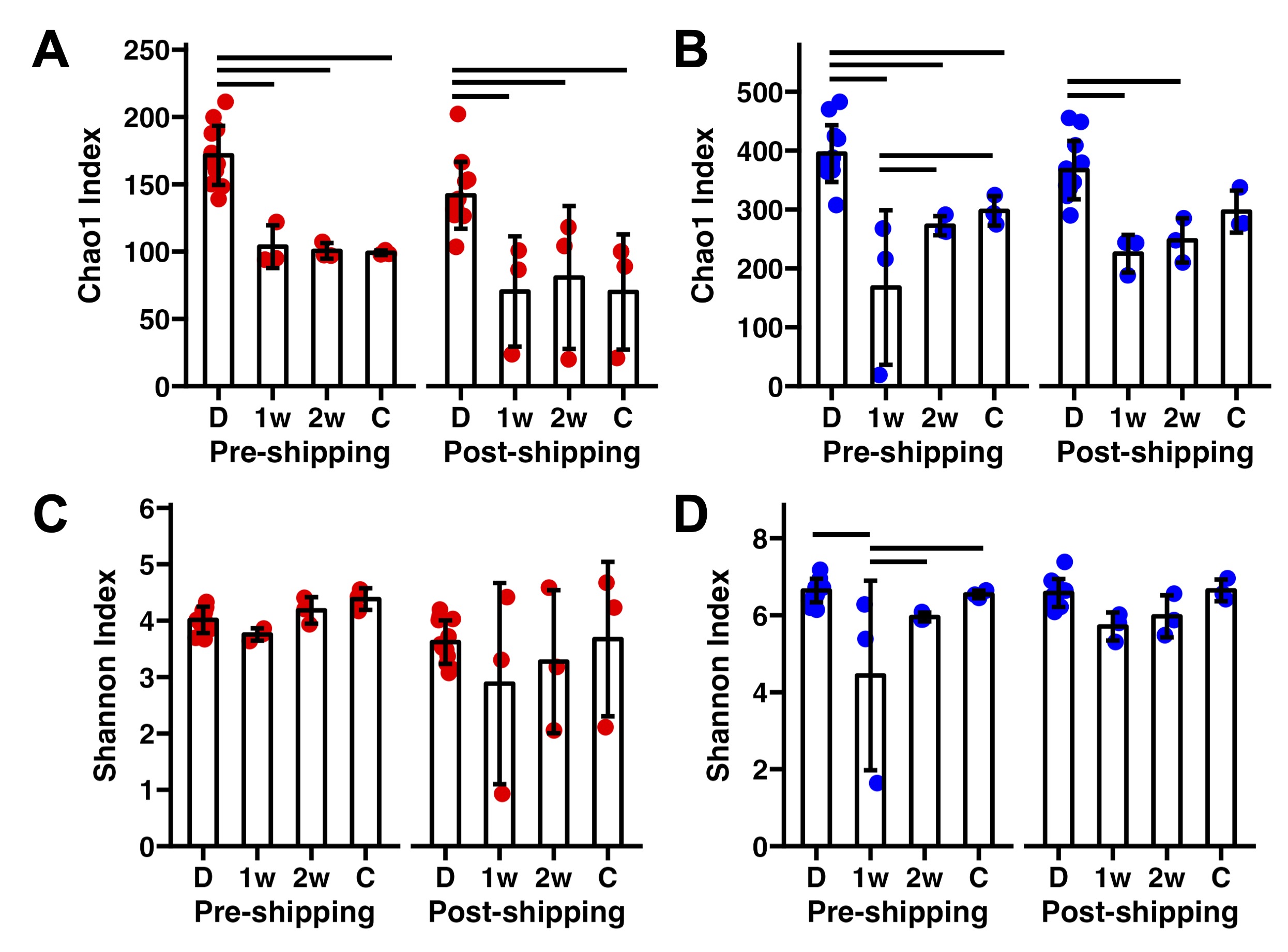

Supplement: Supplemental Material [file KGMR_A_2363858_SM6292.zip › figures/Figure2.jpg]

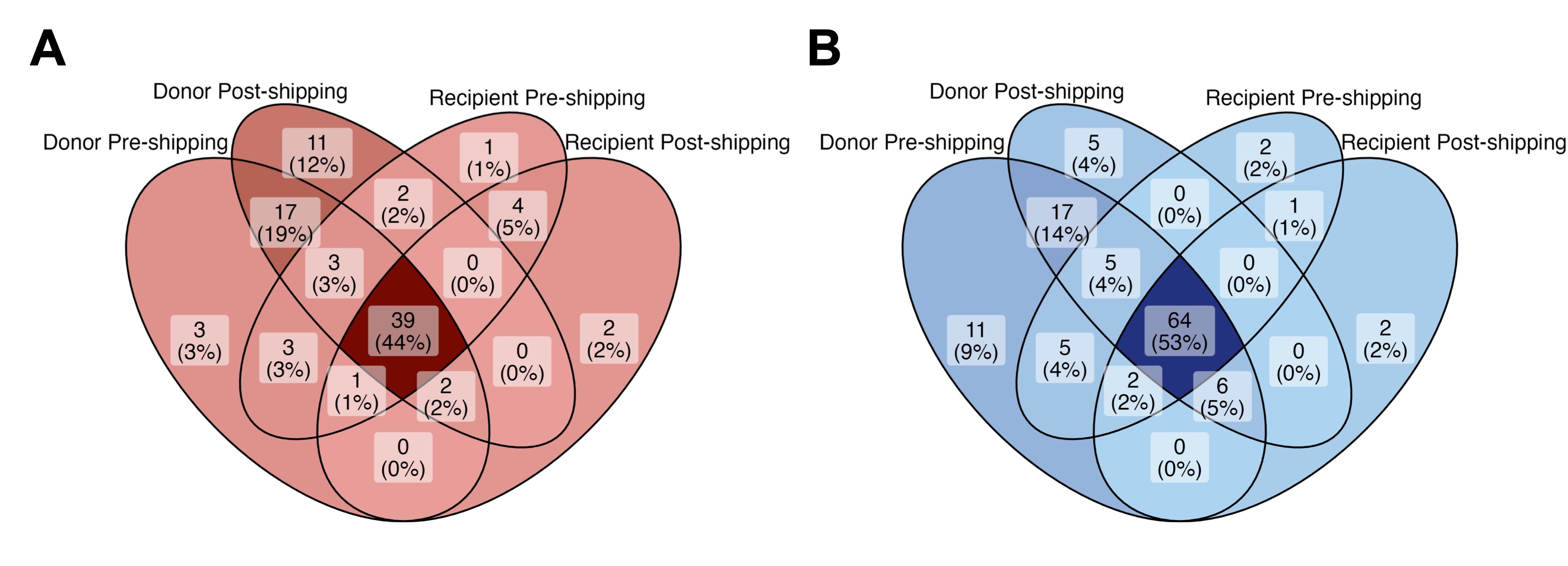

Supplement: Supplemental Material [file KGMR_A_2363858_SM6292.zip › figures/FigureS3.jpg]

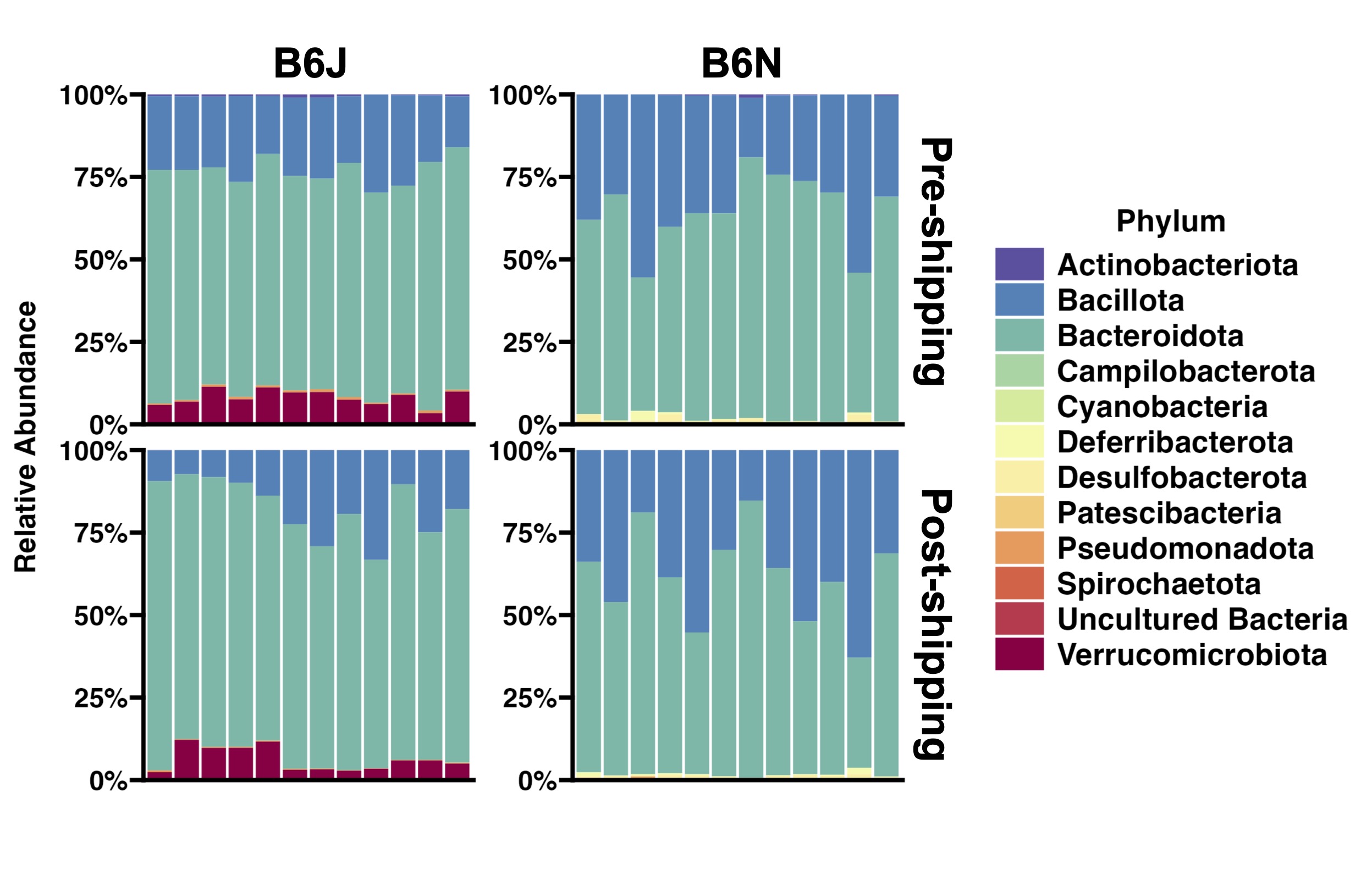

Supplement: Supplemental Material [file KGMR_A_2363858_SM6292.zip › figures/FigureS2.jpg]

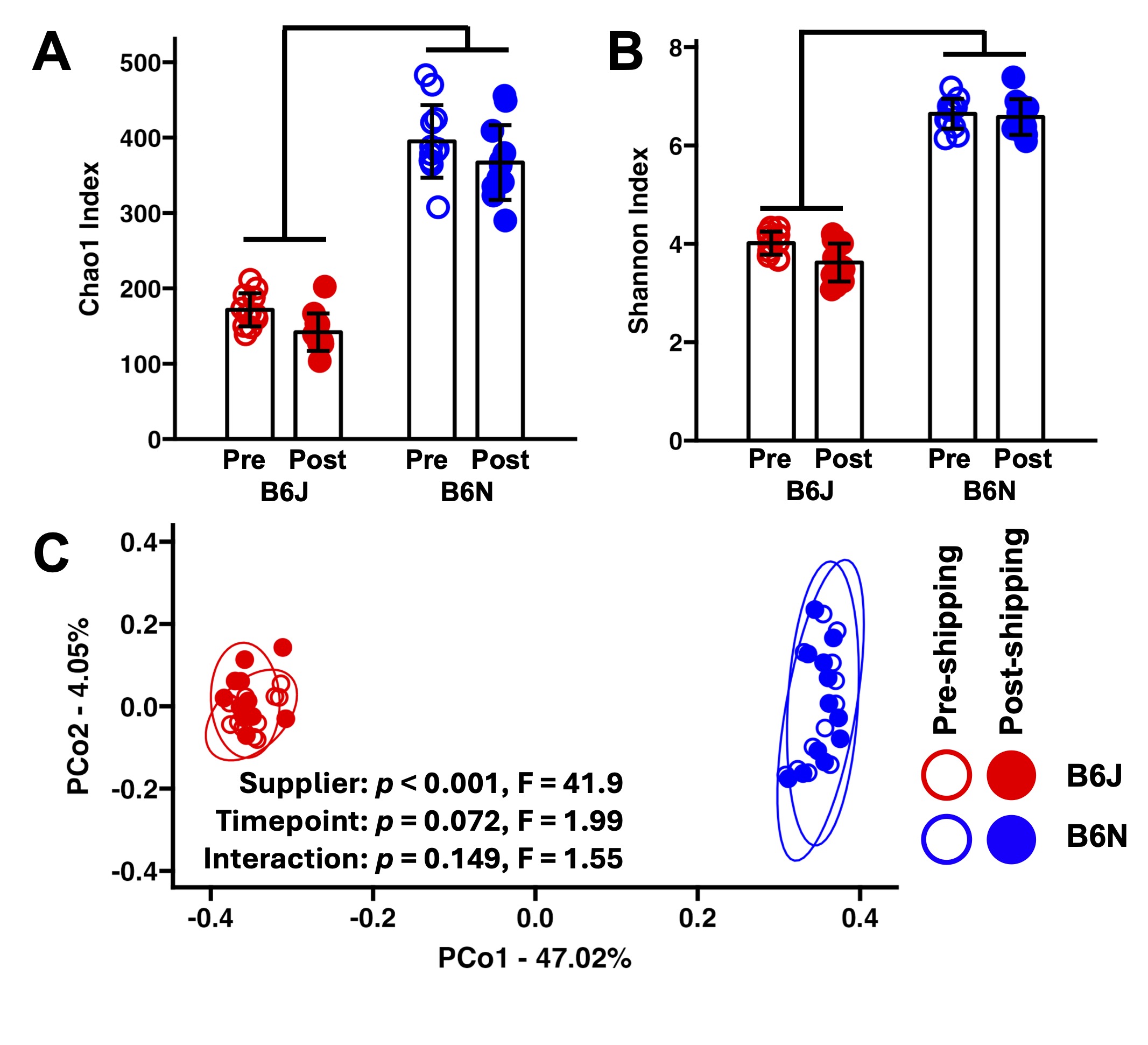

Supplement: Supplemental Material [file KGMR_A_2363858_SM6292.zip › figures/Figure1.jpg]

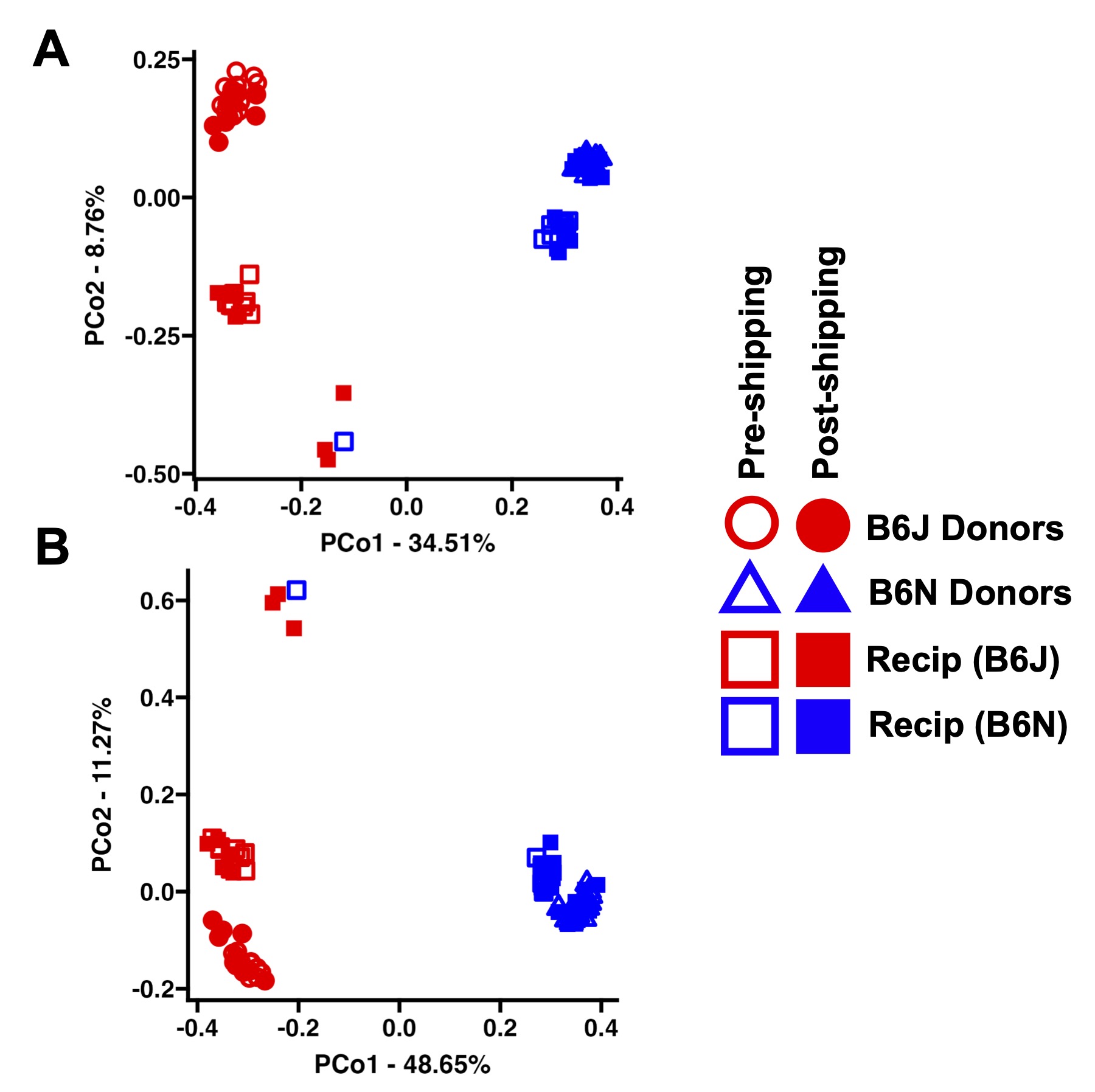

Supplement: Supplemental Material [file KGMR_A_2363858_SM6292.zip › figures/FigureS4.jpg]
